# Fluorescence Output Enhancement of Ce^3+^:YAG Transparent Ceramics by Eutectic Soldering Packaging

**DOI:** 10.3390/ma18051081

**Published:** 2025-02-28

**Authors:** Xuezhuan Yi, Qinglin Sai, Yanna Tian, Renjie Jiang, Mingqin Li

**Affiliations:** Shanghai Institute of Optics and Fine Mechanics, Chinese Academy of Sciences, Shanghai 201800, China; yxz1130@siom.ac.cn (X.Y.); tianyanna@siom.ac.cn (Y.T.); jiangrj@shanghaitech.edu.cn (R.J.); limq@siom.ac.cn (M.L.)

**Keywords:** Ce^3+^:YAG transparent ceramics, eutectic welding, luminescence properties, heat dissipation, deep UV light detection

## Abstract

This paper demonstrates the application of eutectic welding to Ce^3+^:YAG transparent ceramics for reliable detection and imaging of UV emission, particularly focusing on demanding conditions, such as high repetition rate, high energy, and high vacuum. A series of Ce^3+^:YAG transparent ceramics with different Ce^3+^ doping concentrations (0.1 at%, 0.3 at%, 0.5 at%, and 1.0 at%) were prepared via vacuum sintering. Their crystal microstructure, luminescence properties, transmittance, and fluorescence lifetime were studied. It was found that the optimal Ce^3+^ doping concentration is 0.3 at%. The measured ultraviolet-to-visible energy conversion efficiency of the 0.3 at% Ce^3+^:YAG transparent ceramics with a thickness of 1.0 mm is 3.9%. Compared with silicone encapsulated Ce^3+^:YAG transparent ceramic samples, the eutectic-soldered samples exhibited excellent resistance to temperature quenching of the luminescence, which indicates that eutectic welding can effectively improve the fluorescence performance of Ce^3+^:YAG transparent ceramics for the application of deep ultraviolet light detection.

## 1. Introduction

Due to absorption by the atmosphere, electromagnetic waves that fall within the wavelength range of 10–200 nm can only travel in a vacuum, earning them the name vacuum-ultraviolet (VUV) light [1,2,3]. In the field of space science, solar wind-induced aurora, magnetic storms, and ionospheric disturbances on Earth can affect human life [4]. We can monitor solar wind changes by detecting VUV photons, and this has become an important driving force for the development of various VUV photodetectors [5]. Ce-doped yttrium aluminum garnet is an excellent scintillation material for detecting VUV photons. For the application scenario in high vacuum, robust packaging with high heat dissipation capacity is required. However, the traditional scintillation device packaging typically uses silicone gel to bond scintillation ceramics, and silicone gel has clear disadvantages, such as low thermal conductivity and poor thermal stability. Because the heat generated in the fluorescent ceramic package and the secondary heat generated during the light conversion process can be substantial, the fluorescent glue layer is prone to severe aging, yellowing, carbonization, and other effects under long-term thermal radiation and light irradiation. This makes it difficult to meet the packaging requirements of scintillation materials and poses a risk of gas release in high vacuum [6,7,8,9]. To address these thermal management issues, significant research effors have been made. Xie et al. proposed an Al_2_O_3_ composite YAG:Ce fluorescent ceramic, showing good crystallinity and high external quantum efficiency. Al_2_O_3_ can reduce birefringence-related scattering, and the thermal conductivity is as high as 18.5 W m^−1^K^−1^ [10]. Chen et al. prepared a YAG:Ce transparent composite ceramic coated with Al film. YAG:Ce shows high thermal conductivity (29.3 W m^−1^K^−1^) due to the combination of short-range heat conduction of Al_2_O_3_ crystallites and long-range heat conduction of Al film [11]. LED packaging usually involves thermal management, and the combination of high thermal conductivity material Cu and YAG ceramics through eutectic welding technology can effectively improve the heat dissipation capacity in high-power LED applications. Eutectic welding is a process that uses eutectic alloy to form a low-melting-point liquid phase at a specific temperature, enabling metallurgical bonding by wetting the substrate material. Here, we use the eutectic welding method to solve the thermal management problem in the package. Eutectic welding adopts metal solder to weld and fix the crystal on the heat sink. The solder is a pure inorganic material and has the advantages of high thermal conductivity, high mechanical strength, etc., which also helps improve the luminescence performance of Ce^3+^:YAG transparent ceramics [12,13,14].

Cerium-doped yttrium aluminum garnet (Ce^3+^-doped Y_3_Al_5_O_12_, hereafter referred to as Ce^3+^:YAG) has attracted considerable attention due to its outstanding optical, thermal, and mechanical properties [15,16,17,18]. In addition, Ce^3+^:YAG is widely used in scientific research and industrial high-energy beam detection due to its excellent scintillation performance [19,20,21,22,23,24]. The strong absorption peak of Ce^3+^:YAG is in the X-ray band and the extreme ultraviolet band, and its fast decay time (about 70 ns) and high light output in the range of 500–600 nm match well with silicon photodiodes (PDs) and photomultiplier tubes (PMTs) [25,26,27,28]. Moreover, the stable physical and chemical properties and high thermal conductivity of Ce^3+^:YAG ceramics make the material robust in harsh environmental conditions [29,30]. Based on the above advantages, Ce^3+^:YAG is widely used in high-performance applications such as scanning electron microscopy (SEM) and photolithography [31]. Compared with single crystals, transparent ceramics have the advantages of short processing cycle, easy fabrication of special and large shapes, etc. [32,33].

In this paper, Ce^3+^:YAG transparent ceramics were fixed to a copper block heat sink by eutectic welding, and their crystal structure (via SEM), photoluminescence (PL) performance, and heat dissipation capability were investigated in detail, particularly their ultraviolet-to-visible energy conversion efficiency.

## 2. Experimental

First, high-purity Al_2_O_3_ (99.999%, Aladdin, Shanghai, China), Y_2_O_3_ (99.999%, Aladdin, China), and CeO_2_ (99.99%, Aladdin, China) commercial powders were weighed according to the nominal (Ce_x_Y_1−x_)_3_Al_5_O_12_ (x = 0.001, 0.002, 0.003, 0.005, and 0.01) formula. Next, the raw materials added with 0.5 wt% ethyl orthosilicate were mixed by ball milling (Planetary Ball Mill Nanjing Bo Yuntong Instrument XGB2, Shanghai, China) in ethanol for 24 h, followed by drying at 80 °C for 48 h. The resulting powder mixtures were first uniaxially pressed into plates at 10 MPa. Then, after cold isostatic pressing (cold isostatic pressing machine LDJ 100/320–300, Shanghai, China) at 210 MPa, the obtained green bodies were sintered in a high-temperature vacuum furnace (vacuum induction furnace/S furnace, Shanghai, China) at 1750 °C for 7 h. The specific experimental process is shown in Figure S1. Finally, the as-prepared transparent ceramic samples were cut into sheets measuring 20 mm × 20 mm × 1 mm.

X-ray diffraction (XRD) patterns were obtained by X-ray diffractometer, over a test range of 2θ = 5–90° (Bruker D8 ADVANCE, Karlsruhe, Germany). The morphologies of the ceramics were measured using a scanning electron microscope (SEM, Waltham, MA, USA) at 10,000× magnification. The photoluminescence (PL) spectra and luminescence decay spectra of the samples were measured by a fluorescence spectrometer (FLS1000, Edinburgh Instruments, Livingston, UK). The in-line transmittance was recorded by spectrophotometer (Shimadzu, SolidSpec-3700i, Kyoto, Japan). The radiant flux and the ultraviolet-to-visible energy conversion efficiency of the Ce^3+^:YAG transparent ceramic samples were measured by a spectrometer within an integrating sphere. The excitation source was a UV LED chip driven by 200 mA@ 5.0 V, where a power of 25 mW of 250 nm UV emission was generated. A hand-held thermal imaging camera (HM-TPH21PRO-3AQF, Shanghai, China) was used to detect the surface temperature of the Ce^3+^:YAG transparent ceramic samples.

In this study, the crystals and copper blocks were welded together by eutectic welding. The specific operation process was as follows: First, a layer of metal nickel film was laid on the crystal, and then the 2 mm thick solder was cut into a wafer smaller than the crystal diameter and placed between the crystal and the copper block. Next, the copper block, weldment, and crystal were transferred on a 900° vacuum weld for 3 h, and slowly cooled to form a Ce: YAG-CU adhesive layer with high strength and high thermal conductivity.

## 3. Results and Discussion

### 3.1. Concentration Optimization Experiment

**Figure 1:**
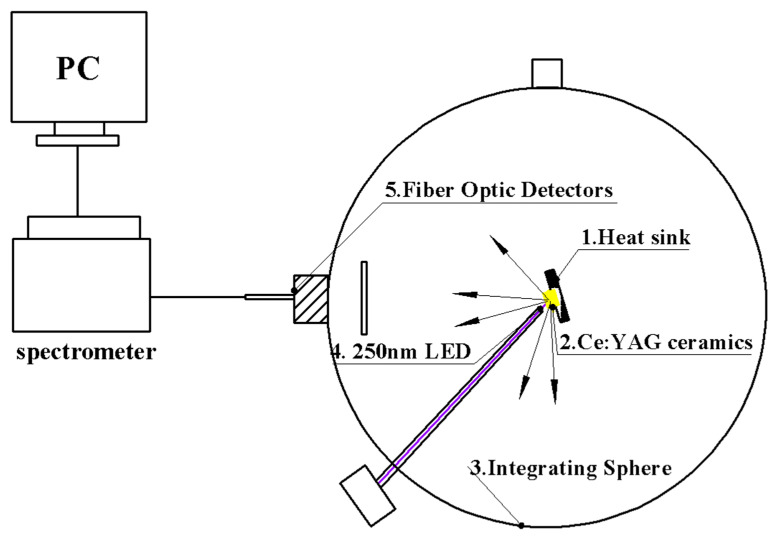
Schematic diagram of the integrating sphere spectrometer for measuring the ultraviolet-to-visible energy conversion efficiency of the Ce^3+^: YAG transparent ceramics.

Figure 1 shows a schematic diagram of the energy conversion efficiency measurement set-up of the Ce^3+^:YAG in the ultraviolet band. During the measurement process, considering the relatively low radiant flux of the ultraviolet LED, the integration time for energy collection was adjusted to 60,000 ms, and the driving current was set to 200 mA.

**Figure 2:**
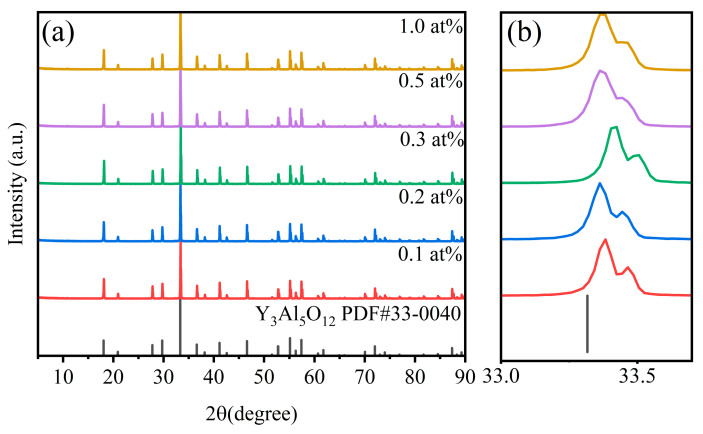
(**a**) XRD patterns of xCe^3+^: YAG (x = 0.1–1.0 at%), (**b**) an enlarged view in the range of 33.0–33.5° of xCe^3+^: YAG (x = 0.1–1.0 at%).

Figure 2a compares the XRD patterns of the sample and the standard PDF card. As shown in Figure 2, the vacuum-sintered ceramic sample is YAG, and all diffraction peaks are consistent with the PDF card (33-0040), and no obvious impurity phase is produced. By comparison, it is found that with the increasing Ce^3+^ doping amount, the diffraction angle of the main crystal plane (420) of the sample shifts to a lower angle. This is because Ce^3+^ with a large ion radius replaces Y^3+^ with a small ion radius, and the interplanar spacing increases. According to the crystal diffraction Bragg equation nλ = 2dsinθ, 2θ becomes smaller, that is, the peak position moves to a small angle, which indicates that Ce^3+^ is successfully doped into the lattice to form a solid solution, as shown in Figure 2b.

**Figure 3:**
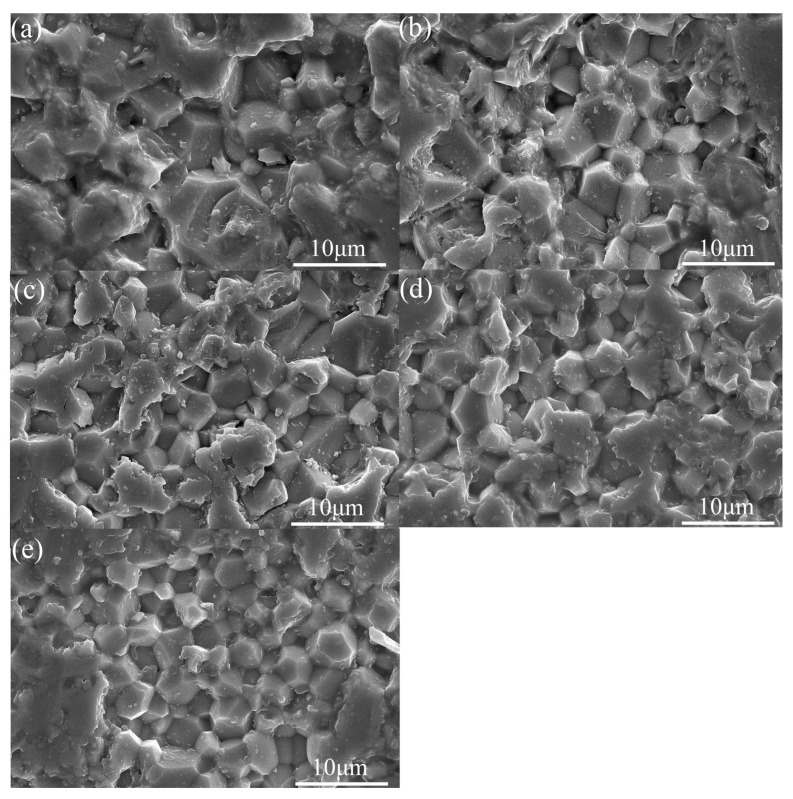
(**a**–**e**) Cross-sectional SEM morphology of the xCe^3+^:YAG (x = 0.1–1.0 at%).

**Figure 4:**
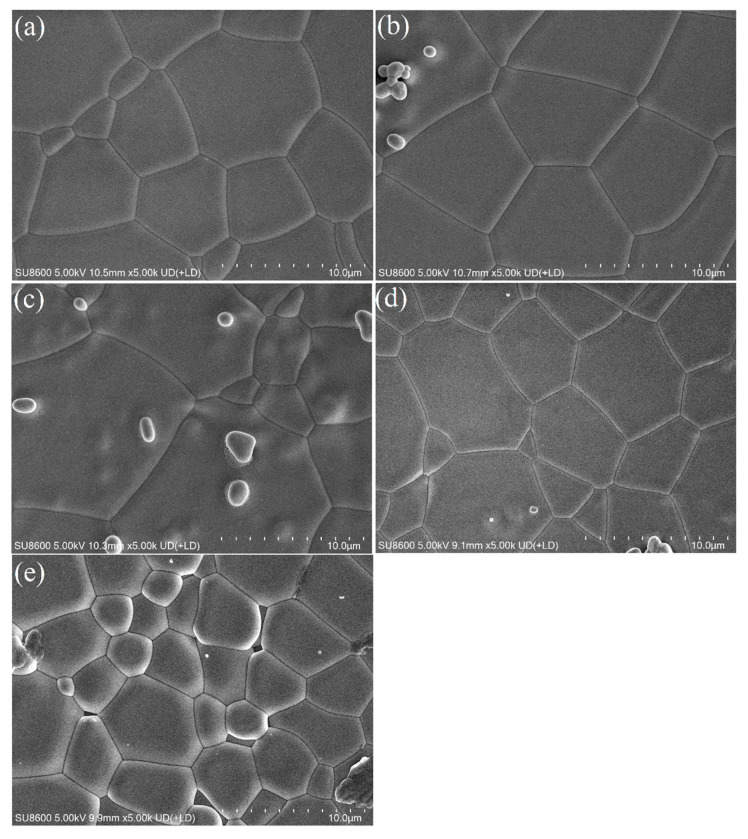
(**a**–**e**) Surface SEM morphology of the xCe^3+^:YAG (x = 0.1–1.0 at%).

Figure 3a–e and Figure 4a–e show the cross-sectional and surface SEM morphology of the sample xCe^3+^: YAG (x = 0.1–1.0 at%). The microstructure of the ceramic sample is dense and the grain boundary is clear. It can be seen that the grain size distribution is uniform, ranging from 3–5 μm, and no obvious pores or fractures are observed. With the increase of Ce^3+^ concentration, the grain size of the sample gradually becomes smaller, indicating that Ce^3+^ can promote the densification of YAG ceramics.

**Figure 5:**
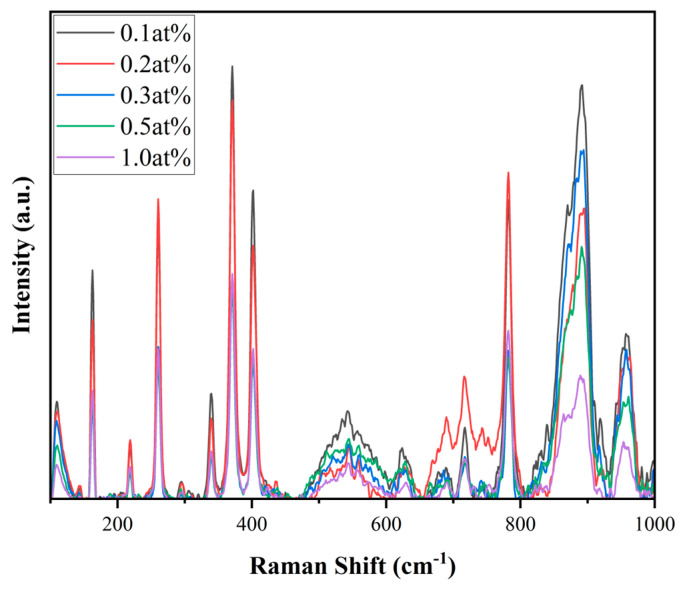
Raman spectroscopy of the xCe^3+^:YAG (x = 0.1–1.0 at%).

As shown in Figure 5, we tested the Raman spectrum of xCe^3+^: YAG (x = 0.1–1.0 at%). In the YAG lattice, the Raman peak at 100–200 cm^−1^ is related to the vibration of metal cations. The peaks at 200–300 cm^−1^ are due to the bending vibrations between O-Al-O and O-Y-O bonds, and the vibrations with frequencies between 300 cm^−1^ and 900 cm^−1^ can be attributed to the symmetrical stretching vibrations of the Al-O bonds [34]. It can be seen that as the doping concentration increases, the peak intensity gradually decreases.

**Figure 6:**
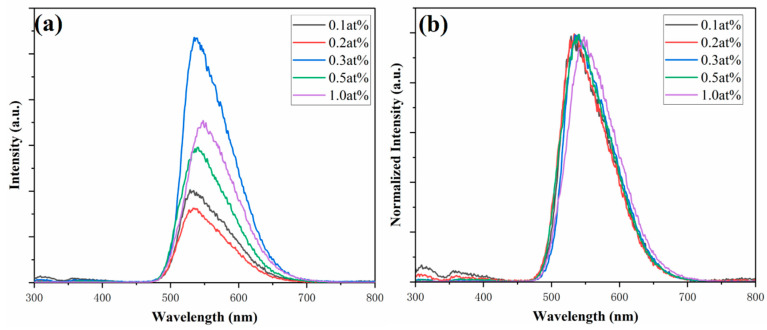
PL spectra (**a**) and normalized PL spectra (**b**) (λ_ex_ = 250 nm) of the xCe^3+^:YAG (x = 0.1–1.0 at%).

Next, the luminescence properties of the samples were studied. As shown in Figure 6a, the PL spectra (λ_ex_ = 250 nm) of the xCe^3+^: YAG (x = 0.1–1.0 at%) were tested, and the emission peak of the samples was at 530 nm. The emission intensity increases with the increase of Ce^3+^ doping concentration and reaches the maximum at x = 0.3 at%. Beyond this doping concentration, the emission intensity decreases due to concentration quenching [35].

It is worth noting that as the Ce^3+^ concentration increases from x = 0.1 at% to x = 1.0 at%, the emission peak of the sample shifts from 530 nm to 550 nm (Figure 6b). This can be attributed to the change in crystal-field splitting (CFS) in the YAG host [36,37]. The CFS strength, *D_q_*, can be written as follows [38,39]:

(1) $$D_{q}=\frac{1}{6}Ze^{2}\frac{r^{4}}{R^{5}}$$

where *Z* is the anion charge, *e* is the electron charge, *r* is the radius of the *d* wave function, and *R* is the bond length. The doping of Ce^3+^ leads to the increase of lattice parameters and the decrease of Ce-O distance. According to Equation (1), *D_q_* around Ce^3+^ will increase, 5d_1_ will decrease, and then Ce^3+^ shows redshift emission.

**Figure 7:**
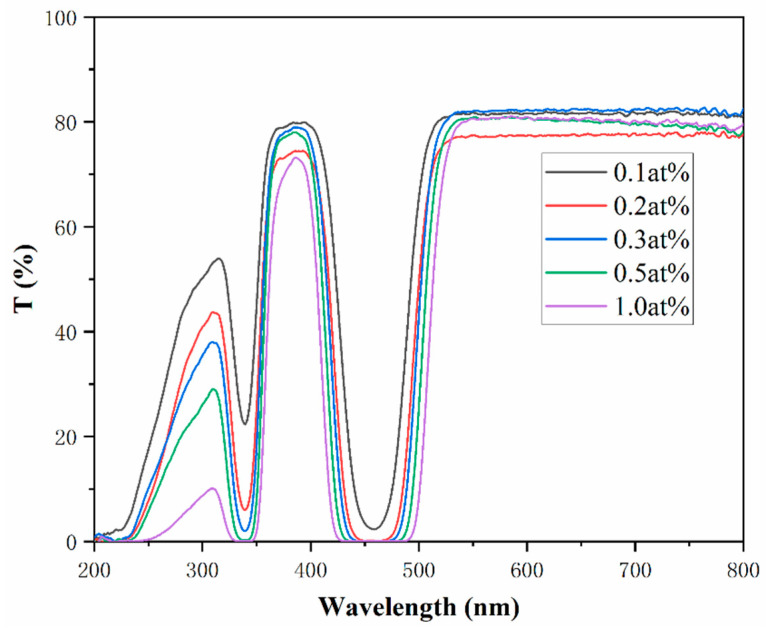
Transmittance curves of the xCe^3+^:YAG (x = 0.1–1.0 at%).

The transmittance curves of the samples were tested and are shown in Figure 7. The samples maintained high transmittance, reaching up to 80%. The lower transmittance in the 300 nm–350 nm and 400 nm–500 nm ranges can be attributed to the absorption of Ce^3+^. In addition, the Ce^3+^ absorption increases with the increases of doping concentration, resulting in a gradual decrease in the transmittance of the above two bands [40].

**Figure 8:**
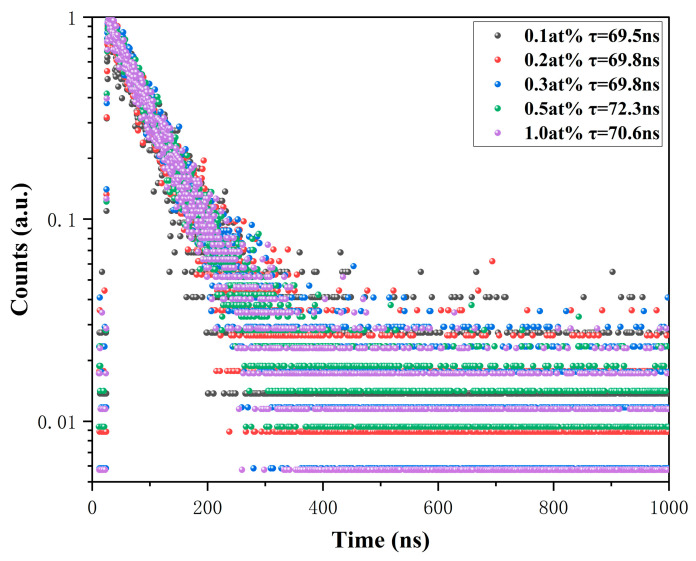
Fluorescence decay curves of the xCe^3+^:YAG (x = 0.1–1.0 at%).

Next, the fluorescence decay curves of xCe^3+^:YAG (x = 0.1–1.0 at%) fluorescent ceramics at 530 nm were tested, which showed a single exponential model, as shown in Figure 8. We obtained the sample decay lifetime by fitting the single exponential decay equation, as shown in Equation (2):

(2) $$I\left(t\right)=A+Bexp\left(-t/\tau \right)$$

We obtained the sample decay lifetime by fitting the single exponential decay equation, as shown in Equation (2), where *A* and *B* are constants, *I* represent the fluorescence emission intensity at time *t*, and *t* represents the fluorescence decay lifetime of Ce^3+^. The fitting results show that as the Ce^3+^ doping concentration increases from x = 0.1 to x = 1, the fluorescence decay lifetimes of the samples are 69.5 ns, 69.8 ns, 69.8 ns, 72.3 ns, and 70.6 ns, respectively [41].

**Figure 9:**
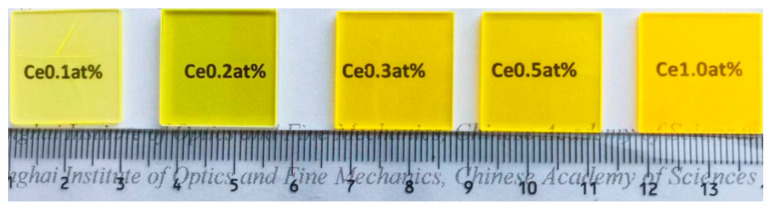
A picture of the Ce^3+^:YAG transparent ceramic samples with different doping concentrations of Ce^3+^.

**Figure 10:**
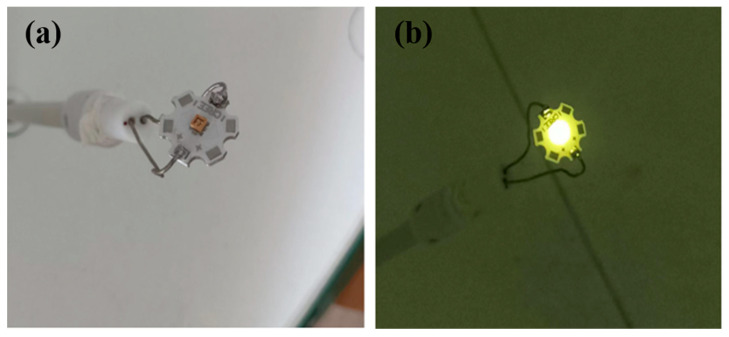
250 nm UV chip inside the integrating sphere (**a**), a picture of the UV chip covering the scintillation ceramics and lighting up (**b**).

All the Ce^3+^:YAG transparent ceramic samples prepared in this experiment are shown in Figure 9. Pictures of the Ce^3+^:YAG transparent ceramic in the integrating sphere with the UV LED chip off/on are shown in Figure 10. Strong yellow emission can be observed for all the Ce^3+^:YAG transparent ceramic samples.

**Figure 11:**
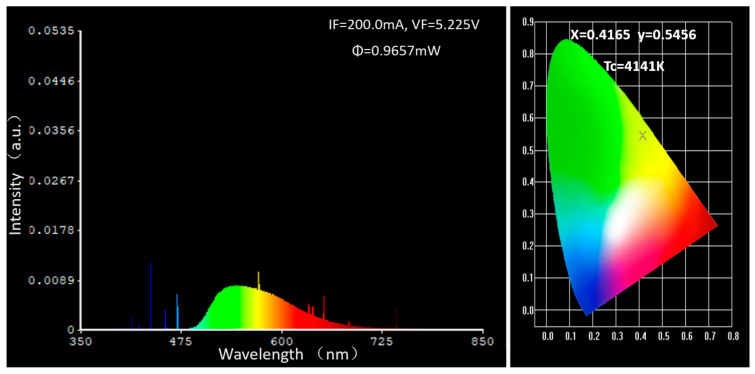
The output power collected by the 0.3 at% Ce:YAG sample in the integrating sphere test system.

The energy collected and conversion efficiency within the integrating sphere are shown in Table 1. With the increase in doping concentration, the output energy shows a trend of first increasing and then slightly decreasing. The conversion efficiency of 0.1–0.3 at% Ce:YAG ceramic shows a significant positive correlation with the change of concentration, indicating an increase in the number of luminescent centers within a certain range, which can effectively improve the probability of fluorescence conversion to ultraviolet energy. The increase of the Ce^3+^ doping concentration from 0.3 to 1.0 at% leads to a gradual decrease in energy conversion efficiency, which may be related to self-absorption under high doping concentration. The output power and spectrum of the 0.3% sample were tested by integrating spheres, as shown in Figure 11.

### 3.2. Eutectic Welding Experiment

According to the energy conversion efficiency measurement results, 0.3 at% Ce:YAG ceramic shows the highest energy conversion efficiency. Therefore, the 0.3 at% Ce:YAG sample was selected as the raw material for the second part of the eutectic welding comparison experiment.

**Figure 12:**
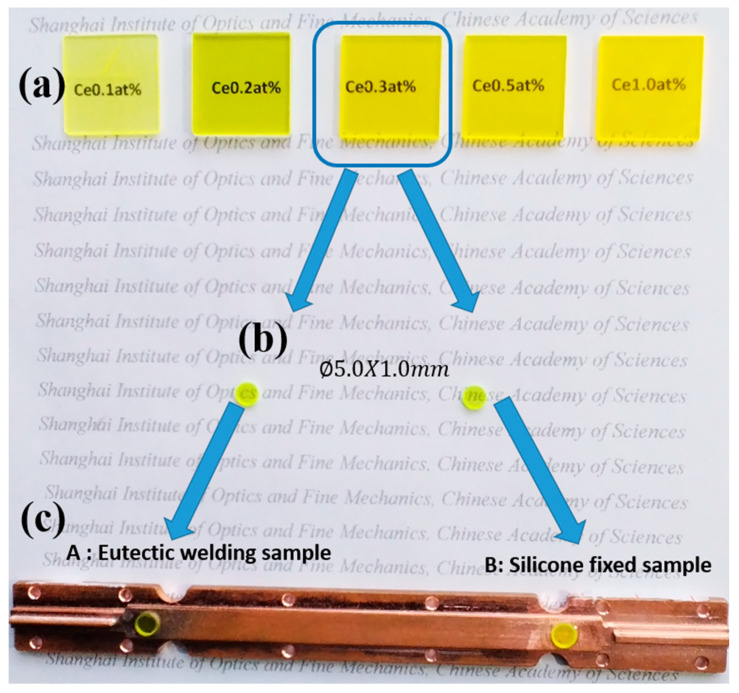
Photographs of the prepared scintillation ceramic series samples (**a**), packaged wafers processed from 0.3 at% samples (**b**), two sets of samples using eutectic welding (A) and silicone fixation (B), respectively (**c**).

The raw materials for eutectic soldering packaging experiments were cut from 0.3 at% Ce:YAG samples. Two sets of experiments, A and B, were designed to study the impact of eutectic soldering on heat dissipation. Specifically, Group A was packaged using high thermal conductivity silver copper solder eutectic soldering, while Group B used traditional solid crystalline silicone as packaging material, as shown in Figure 12.

**Figure 13:**
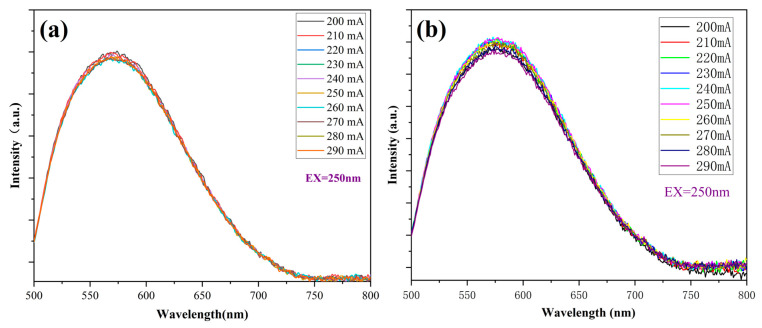
The photoluminescence (PL) spectra of scintillation ceramics under different driving currents: eutectic welding sample (**a**), silicone fixed sample (**b**).

The HASS-2000 integration sphere system was used to evaluate the luminescence stability and conversion efficiency of scintillation ceramics by measuring the integration intensity of their fluorescence spectra under different injection currents. Figure 13 shows that as the injection current of the ultraviolet LED gradually increases from 100 mA to 300 mA, the corresponding output ultraviolet energy also gradually increases, resulting in a rise in the temperature of the scintillation ceramic. At the same time, due to the increase in operating current, the thermal effect of the LED chip itself is obvious. Two groups of samples, A and B, obtain the same amount of heat under the same conditions. The study found that as the injected current increased, the fluorescence spectrum of group A did not show an obvious downward trend, while the reference group B samples showed an obvious downward trend. These results indicate that the scintillation ceramic encapsulated by eutectic welding has a significant thermal conductivity advantage.

**Figure 14:**
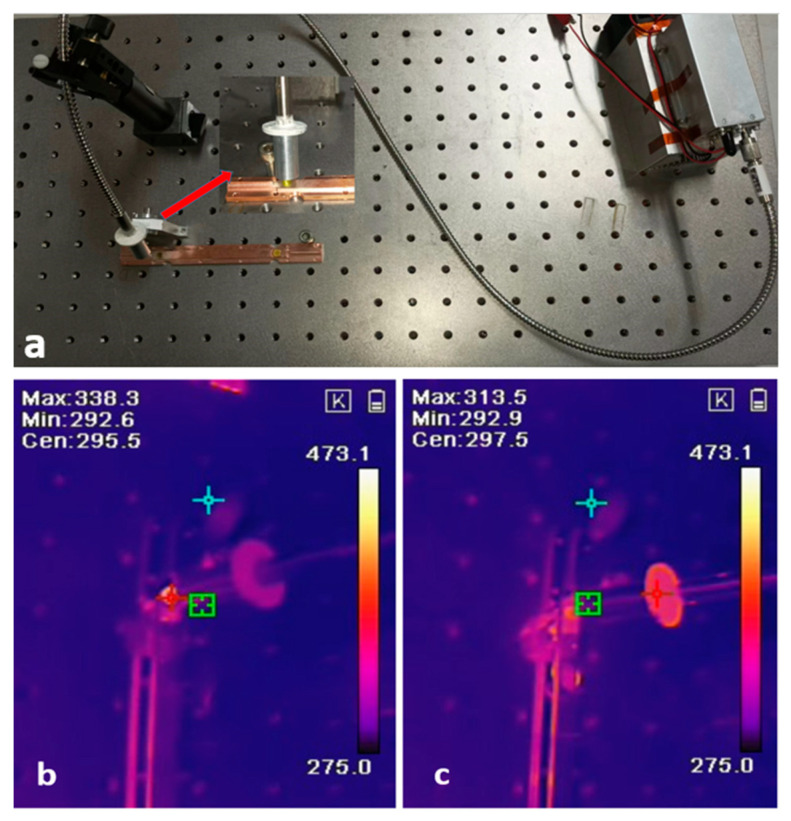
Actual picture of heat dissipation performance test device (**a**) and infrared thermal imaging images of two groups of scintillation ceramics under high-power laser excitation: silicone fixed sample (**b**), eutectic welded sample (**c**). Arrows are enlargement of local images.

In order to more intuitively observe the heat dissipation effect of samples in groups A and B, a high-power 450 nm semiconductor laser was used as the excitation light source to focus the output laser on the samples of groups A and B. After stabilizing for 3 min, the thermal imaging camera was used to directly capture the temperature field distribution of the two groups. As shown in Figure 14, in the high-temperature region, the maximum working temperature of the sample with the eutectic welding method is 313.5 K. As the reference group, Group B showed a working temperature as high as 338.3 K. Obviously, the eutectic welding method has a significant advantage in heat dissipation.

## 4. Conclusions

A series of scintillation ceramic samples were prepared, and the crystal structure and luminescence properties of the samples were comprehensively studied. SEM tests show that the samples have a dense crystal structure with a grain size of 3–5 μm; the sample has the highest luminescence intensity at x = 0.3 at%, and the transmittance at 500–600 nm is 80%, corresponding to a fluorescence lifetime of 69.8 ns. Importantly, it was found that the highest energy conversion of 0.3 at% Ce^3+^ doped scintillation ceramics can reach 3.86%. At the same time, a series of experiments on eutectic welding and silicone fixed packaging were conducted, and the PL spectra of the selected samples were tested at different currents. Experimental results show that the use of eutectic welding technology can significantly reduce the temperature of the scintillation ceramic. Therefore, the use of eutectic welding technology as a package for scintillation ceramics has great application value. However, the fluorescence intensity of eutectic packaged samples is low at present, and future improvements will focus on optimizing raw materials and refining the preparation process.

## Figures and Tables

**Table 1:** Output energy and ultraviolet-to-visible energy conversion efficiency of all the Ce:YAG transparent ceramic samples under 25 mW UV LED excitation.

Doping Concentration of Ce	0.1 at%	0.2 at%	0.3 at%	0.5 at%	1.0 at%
Output energy (mW)	0.3002	0.4022	0.9657	0.8011	0.7160
Energy conversion efficiency	1.20%	1.61%	3.86%	3.20%	2.86%

## Data Availability

The original contributions presented in this study are included in the article/Supplementary Material. Further inquiries can be directed to the corresponding author.

## Supplementary Materials

The following supporting information can be downloaded at: https://www.mdpi.com/article/10.3390/ma18051081/s1, Figure S1: Flowchart of the preparation of Ce^3+^:YAG ceramics.
