# Supplementary material for: Fluorescence Output Enhancement of Ce3+:YAG Transparent Ceramics by Eutectic Soldering Packaging
Source: Materials (Basel). 2025 Feb 28;18(5):1081. doi: 10.3390/ma18051081 (PMC11901220; doi:10.3390/ma18051081)
Supplement: Supplementary file 1 [file materials-18-01081-s001.zip › materials-3427235-supplementary.pdf]

Fluorescence output enhancement of Ce^{3+}:YAG transparent ceramics by eutectic soldering packaging

Xuezhuan Yi, Qinlin Sai *, Yanna Tian, Renjie Jiang and Mingqin Li

Shanghai Institute of Optics and Fine Mechanics, Chinese Academy of Sciences, Shanghai 201800, China

* Correspondence: author: saiql@siom.ac.cn (Q. Sai)

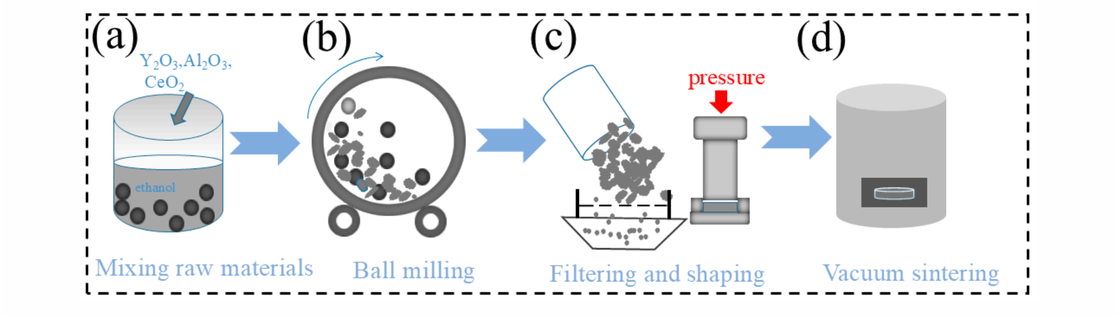

Figure S1. Flowchart of the preparation of Ce: YAG ceramics.
